# Integrating longitudinal metabolite profiles improves phenotype prediction in pigs in a trait- and timepoint-dependent manner

**DOI:** 10.1186/s12711-026-01092-3

**Published:** 2026-10-13

**Authors:** Quazi Abir Hassan Roddur, Dingzhen Liang, Jiayi Qu, Eula Regina Carrara, Daniela Lourenco, Bruno Valente, Ching-Yi Chen, Justin Holl, Hao Cheng

**Affiliations:** 1 University of California, 1 Shields Ave, Davis, CA 95616 United States; 2 University of Georgia, 100 Green St, Athens, GA 30602 United States; 3 PIC North America, 100 Bluegrass Commons Blvd, Ste 2200, Hendersonville, TN 37075 USA

## Abstract

**Background:**

Accurate prediction of phenotypes is essential for assessing the actual productive performance of individual pigs, particularly for traits that are costly or difficult to measure directly. This study investigated the potential of integrating individual-level blood serum metabolite profiles sampled at two developmental stages (10-week and 20-week) into phenotype prediction models for five economically important traits: average daily feed intake (DFI), feed conversion ratio (FCR), backfat thickness (BF), test daily gain (TDG), and loin depth (LD). Using a BayesC modeling framework, we analyzed 1,637 pigs from a single purebred population with complete phenotype, genotype, and metabolite-profile data. We evaluated seven models, including a genotype-only model (G), metabolite-only models using metabolite-profile data from 10-week (M1), 20-week (M2), or both timepoints (M1+M2), and combined models integrating genotypes with metabolite-profile data (G+M1, G+M2, G+M1+M2).

**Results:**

Heritability estimates were generally low to moderate, ranging from 0.08 to 0.23 for the 10-week metabolite profiles and from 0.07 to 0.26 for the 20-week metabolite profiles. Prediction accuracy of phenotypes consistently improved when metabolite-profile data were integrated into prediction models, although the magnitude of improvement varied depending on the trait and the timepoint of metabolite sampling. Prediction accuracy increased from 0.33 (G) to 0.42 (G+M2; G+M1+M2) for DFI, and from 0.27 (G) to 0.32 (G+M2; G+M1+M2) for FCR. The latter two models also delivered the largest gains over G for BF (from 0.39 to 0.46) and TDG (from 0.30 to 0.32). However, LD benefited the most when both 10-week and 20-week metabolite profiles were combined (G+M1+M2: 0.44 compared to G: 0.42).

**Conclusions:**

Across all traits, models combining genotype data with metabolite profiles from one or multiple timepoints achieved the highest or equally high phenotype prediction accuracies compared to the genotype-only model, reflecting complementary biological insights captured by metabolite profiles. These findings highlight the potential value of metabolite-profile data as an intermediate omics layer to enhance phenotypic prediction, particularly when integration strategies are tailored to trait-specific biology and sampling timepoints.

**Supplementary Information:**

The online version contains supplementary material available at https://doi.org/10.1186/s12711-026-01092-3.

## Background

Accurate prediction of phenotypic performance is essential for improving selection and management decisions in livestock production, thus accelerating productive performance [1, 2]. Approaches that leverage genotypes and phenotypes from relatives have become standard practice in animal breeding [3, 4] since the proposal of genomic prediction using simulated data [5]. In pig breeding, traits of economic importance, such as feed efficiency, growth, and reproduction, are often expensive, labor-intensive, or challenging to measure directly in selection candidates [6, 7]. Therefore, there is considerable interest in identifying biologically informative intermediate phenotypes that can be reliably measured and integrated into selective breeding programs [8–10].

Metabolite-profile data, which encompass the comprehensive profiling of small molecules in biological systems, offer a dynamic snapshot of an organism’s physiological and biochemical state [11]. Blood metabolite-profile data have shown promise as intermediate phenotypes because they reflect underlying biochemical pathways associated with economically important traits, including feed efficiency [12, 13]. Since the metabolome displays both genetic and environmental influences, it offers a unique opportunity to bridge the gap between genotypes and complex phenotypes in prediction models [14]. The integration of metabolite-profile data into genetic evaluations has been previously explored in crops such as barley [15] and livestock such as pigs [16]. Specifically in the pig application, the study focusing on average daily gain in Duroc pigs has reported small increases in prediction accuracy of breeding values when including metabolite-profile data collected at the end of the testing period [16].

However, the timing of metabolite sampling plays a crucial role [17], and the effects of metabolites on phenotypes may vary across different traits [18]. Studies have shown that metabolite-profile data differ significantly by age, breed, and feeding strategy, indicating the value of collecting omics data at multiple physiological stages [19, 20]. Additionally, the limited improvement in prediction accuracy might stem from a mismatch between the time of metabolite sampling and the time-integrated nature of average daily gain, highlighting the importance of temporal dynamics in omics-based prediction [16].

In our study, we address this by analyzing serum metabolite-profile data collected at two developmental stages, 10 and 20 weeks of age. This phase span is particularly important, as pigs undergo rapid growth and metabolic transitions during this time, which profoundly influence traits such as feed efficiency and carcass composition. By evaluating five economically important traits, including average daily feed intake (DFI), feed conversion ratio (FCR), backfat thickness (BF), test daily gain (TDG), and loin depth (LD), we provide a more comprehensive assessment of the potential value of metabolite-profile data in pigs. We hypothesize that integrating individual-level metabolite-profile data collected at multiple growth stages into prediction models will improve phenotype prediction accuracy compared to using genomic data alone. We further anticipate that the degree of improvement will vary according to the specific trait and timing of metabolite sampling. We also expect that incorporating metabolite-profile data will consistently increase the signals available to prediction models without compromising accuracy.

## Methods

### Dataset

#### Data collection

Data were provided by PIC (a Genus company, Hendersonville, TN, USA). The pigs included in this study were randomly allocated to pens equipped with feeders that allowed individual feed intake to be recorded. Feed intake data were collected from 10 to 20 weeks of age. Pigs were provided ad libitum access to feed. The first three days were excluded as a training period. Pigs were weighed before the start of this 10-week period and again at 20 weeks of age. Ultrasound measurements of BF and LD were also taken at 20 weeks of age. These records were used to define the traits analyzed in this study: DFI (measured from 10-week to 20-week span), FCR (measured from 10-week to 20-week span), BF (measured at 20-week), TDG (measured from 10-week to 20-week span), and LD (measured at 20-week). Blood samples were also collected at 10 and 20 weeks of age, at approximately 10:00 a.m. Serum extracted from these samples was analyzed using 1 H NMR (proton nuclear magnetic resonance) spectroscopy, generating individual metabolite profile data. Individual ear punch tissue samples were collected for genotyping. All genotypes were imputed to a medium-density panel of 37k markers using AlphaImpute [21]. In total, records from 2,101 pigs were collected for this study.

#### Data availability

The metabolite-profile data were obtained from blood serum samples collected at two timepoints, 10 and 20 weeks of age. The 10-week sampling occurred during the nursery phase, and the 20-week sampling occurred during the finishing phase. Blood samples were taken and stored under controlled conditions at −80 °C to preserve the stability of metabolites prior to analysis. The 1 H NMR spectra of the samples were recorded on a Bruker AV-III 600 MHz spectrometer, which is certified for the In Vitro Diagnostic research (IVDr) analysis, using a BBI probe, and the standardized experiments were conducted at 310 K. The readings were automatically preprocessed by Bruker software, which performed essential steps such as zero-filling to enhance resolution, apodization to improve signal-to-noise ratio, Fourier transformation to convert time-domain signals to frequency-domain spectra, phase correction and baseline alignment, and spectrum calibration for consistent peak referencing. The comprehensive metabolite-profile data were generated using Bruker IVDr Quantification in Plasma/Serum B.I.Quant-PS™ and Bruker IVDr Lipoprotein Subclass Analysis B.I.LISA™ reports. These advanced quantification platforms enabled automated identification and absolute concentration measurements of a wide range of serum metabolites. The resulting profile included a diverse set of metabolites such as amino acids and their derivatives, ethanol, carboxylic acids, keto acids, sugars, and lipoprotein fractions, among others, capturing a broad overview of each animal’s metabolic state at the time of sampling. The raw metabolites dataset included a total of 149 metabolites, composed of 112 lipoproteins and 37 small-molecule metabolites.

#### Data quality control and processing

Quality control (QC) procedures were applied to all datasets used in this study. For metabolite-profile data, metabolites measured in less than 80% of the original 2,101 individuals were excluded. This resulted in a final dataset comprising 78 lipoproteins and 17 small-molecule metabolites, totaling 95 metabolites. For genotype data, standard quality control procedures were applied, including filtering out SNPs with a minor allele frequency (MAF) below 0.01, resulting in 37,199 SNPs retained for analysis. After these QC steps, the final dataset included 1,637 individuals who had complete records for both 10-week and 20-week metabolite-profile data, all five phenotypic traits, and genotype data.

To account for technical variation, metabolite measurements for the 10-week and 20-week datasets were corrected separately for batch effects before downstream analyses. Batch effects were estimated using a linear mixed model that included batch and birth year-month as fixed effects, and litter and additive genetic effects as random effects. Batch effect-corrected metabolite values were obtained by subtracting the estimated batch effects from the observed metabolite measurements.

### Model for analysis

#### Heritability estimation

Heritability for each metabolite was estimated separately in the 10-week and 20-week datasets using the software tool JWAS [22]. Each metabolite was analyzed as a univariate quantitative trait using a linear mixed model that included an intercept, batch, and birth year-month as fixed effects, and litter and additive genetic effects as random effects. Variance components for additive genetic and residual effects were estimated using a Bayesian approach (BayesC). Heritability was calculated as the proportion of phenotypic variance attributable to the additive genetic effect.

For the five traits (DFI, FCR, BF, TDG, and LD), the proportion of phenotypic variance attributable to genotype and metabolite components was evaluated using linear mixed models analogous to those described above. The models included litter as a random effect, an intercept, and trait-specific fixed effects (birth year-month when applicable), with genomic and/or metabolite predictors fitted according to the model specification.

#### Statistical methods

For each trait, we evaluated several linear mixed models that incorporated genotypes and/or metabolite profiles collected at different timepoints, as shown in Table 1. Prior distributions of regression coefficients were specified according to the BayesC method, using two separate sets of hyperparameter values for genotype and metabolite profiles [23]. When only genotypes were included, the prediction model is expressed as:

1 $$\boldsymbol{y = W\theta + X\beta + e}$$

**Table 1:** Overview of the seven predictive models constructed using different combinations of genotype and metabolite-profile data

Model Name	Genotype	Metabolites (10-week)	Metabolites (20-week)
G	Yes	No	No
M1	No	Yes	No
M2	No	No	Yes
M1+M2	No	Yes	Yes
G+M1	Yes	Yes	No
G+M2	Yes	No	Yes
G+M1+M2	Yes	Yes	Yes

Here, ‘Yes’ indicates inclusion and ‘No’ indicates exclusion

where **y** is the *n × 1* phenotype vector (*n* = 1,637 individuals); *θ * is the vector of fixed effects and $\boldsymbol{W}$ is its corresponding design matrix; $\boldsymbol{X}$ is the *n × p* genotype matrix (*p* = 37,199 SNPs), coded as 0, 1, 2 corresponding to zero, one, or two copies of the reference allele, respectively; **β** is the *p × 1* vector of marker effects; and $\boldsymbol{e}$ is the *n × 1* vector of residual variance for each individual with the distribution of $\boldsymbol{e \sim N\left( {0,I\sigma _{e}^2} \right)}$.

In the BayesC framework, marker-effect estimation is based on a biologically meaningful assumption that only a subset of all genomic variants has non-zero effects, while the majority do not influence the phenotype. The model, therefore, infers a null-effect probability, π, which reflects the proportion of SNPs whose effects are zero. For the remaining SNPs, their effect magnitudes are assumed to follow a normal distribution with mean 0 and variance $\boldsymbol{\sigma _\beta ^2}$. Thus, the effect of SNP j is modeled as a mixture distribution comprising a point mass at zero and a normal distribution as expressed as:

2 $$\beta_j \sim \begin{cases} \mathcal{N}(0, \sigma_\beta^2), & \text{with probability } 1-\pi,\\ 0, & \text{with probability } \pi. \end{cases}$$

Metabolite-profile data were included as additional random regressors. When metabolites from 10-week and 20-week were analyzed together, the model became:

3 $$\boldsymbol{y = W\theta + X\beta + {Z_1}{u_1} + {Z_2}{u_2} + e}$$

where variables $\boldsymbol{y,W\theta ,{\rm{}}X\beta ,{\rm{}}}$ and $\boldsymbol{e{\rm{}}}$ are the same as those in the conventional BayesC model as described above, $\boldsymbol{{Z_1}}$ and $\boldsymbol{{Z_2}}$ are the matrices of metabolites from weeks 10 and 20, respectively, where each matrix entry in the *i*^th^ row and *j*^th^ column represents the *j*^th^ metabolite feature for individual *i*, and $\boldsymbol{{u_1}}$ and $\boldsymbol{{u_2}}$ are their corresponding random effect vectors. For traits BF, DFI, and TDG, litter was included as a random effect and birth year-month was included as a fixed effect, whereas for FCR, and LD, only litter was included as a random effect. The choice of including random and fixed effects in the models for each trait was based on their optimal prediction performance in the baseline genotype-only (G) model.

Gibbs samplers were applied to obtain samples of the joint posterior distributions of components within each model. Samples were saved every 5th iteration, yielding 1000 posterior samples after 5000 total iterations. Convergence was verified by visual inspection of trace plots for key parameters.

#### Validation method

Prediction accuracy was evaluated as the Pearson correlation between the predictions and adjusted phenotypes in a testing set. Model performance was compared across the seven models. To evaluate prediction accuracy, the full dataset was randomly divided into a training set (80%) and a testing set (20%). We ensured that all levels of litter and birth year-month were observed in both training and testing sets. Model parameters were estimated using the training set and used to predict adjusted phenotypes for individuals in the testing set. This procedure was repeated for 20 replicates.

**Fig. 1:**
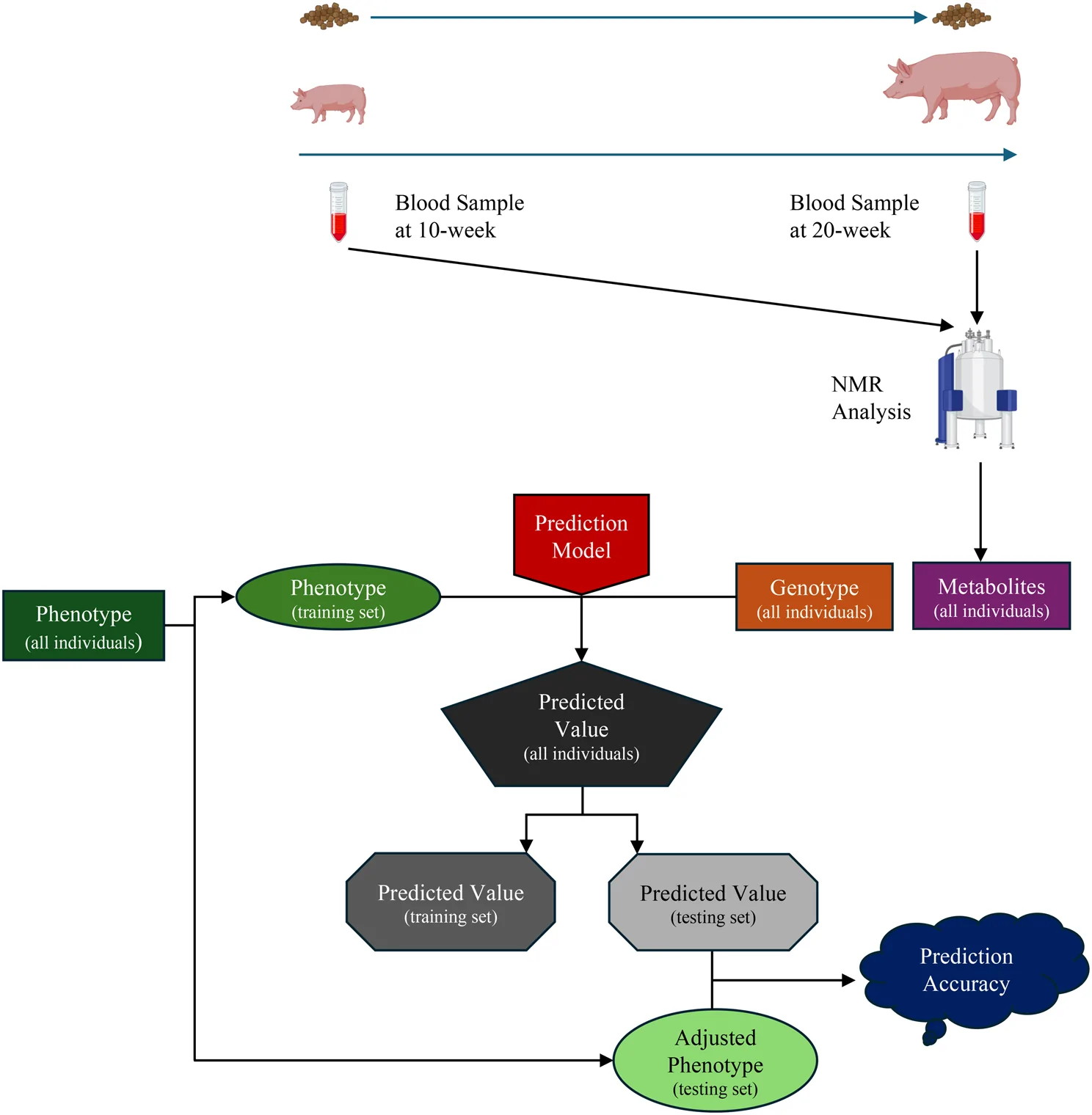
Detailed workflow from data collection to prediction accuracy estimation

Figure 1 illustrates the whole process, including the metabolite-profile data collection procedure and implementation of these datasets in the prediction models.

## Results

### Heritability estimation of metabolites

To assess the genetic basis of serum metabolites variation, we estimated the heritability (h^2^) of 95 metabolites at 10-week and 20-week. The goal of this analysis was to evaluate whether these metabolites are heritable and to quantify their heritability.

**Fig. 2:**
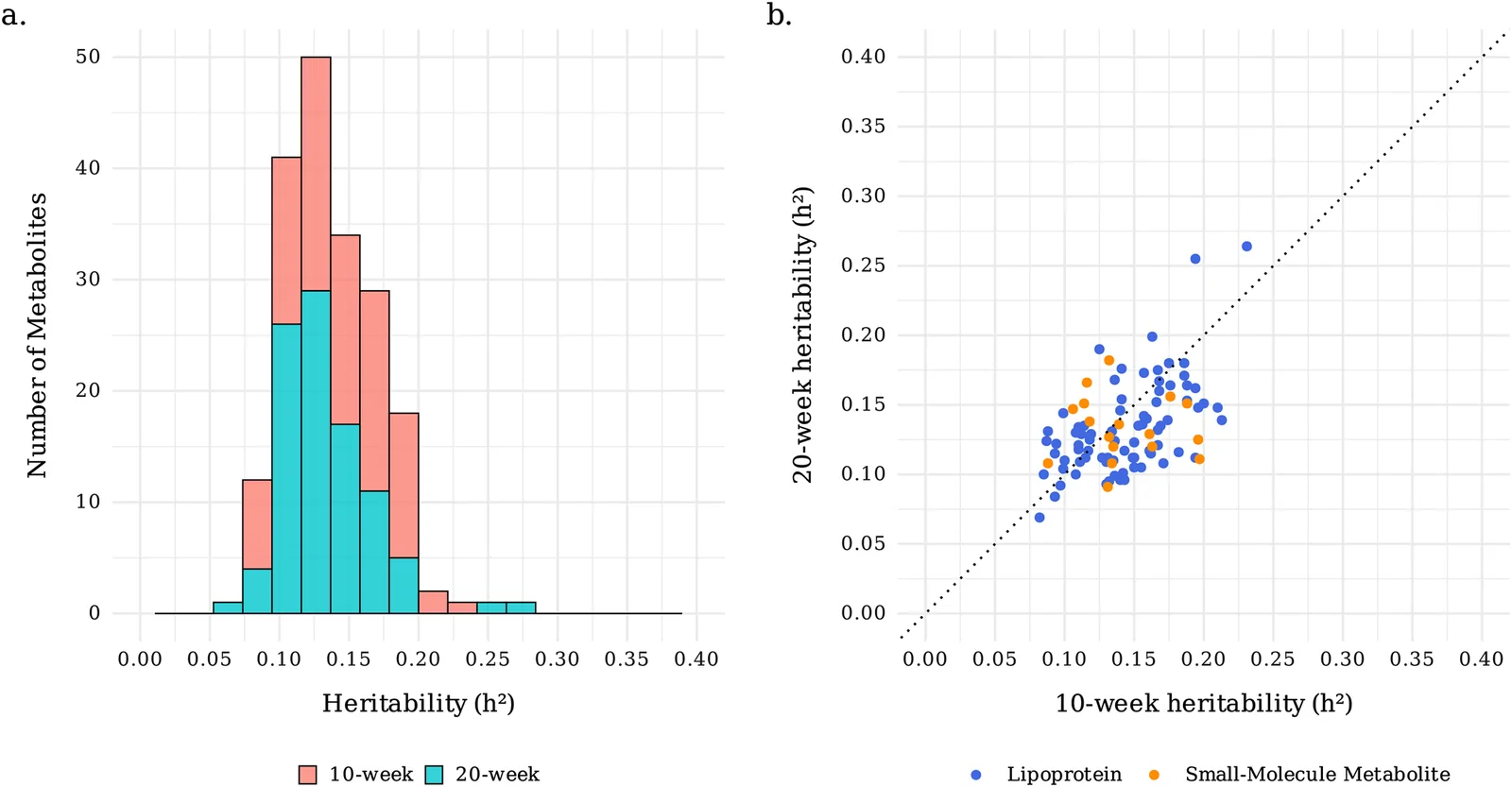
Heritability estimates of metabolites. (**a**) Distribution of heritability estimates for metabolites measured at 10-week and 20-week. (**b**) Scatter plot comparing heritability estimates for individual metabolites between the 10-week and 20-week sampling points. Points above the diagonal line indicate higher heritability at 20-week, whereas points below suggest higher heritability at 10-week

Figure 2a shows the distribution of h^2^ estimates for serum metabolites collected at 10 and 20 weeks of age. Most 10-week estimates (reddish bars) ranged from 0.08 to 0.23, whereas 20-week estimates were distributed over a slightly wider range, from 0.07 to 0.26. Figure 2b compares h^2^ estimates of individual metabolites across the two stages.

### Proportion of variance explained by genotype and metabolite components

Table 2 summarizes the proportion of phenotypic variance explained by genotype and metabolite components across different models. In general, incorporating metabolite information improved or maintained the variance explained compared to genotype-only models, although the magnitude of improvement varied across traits and metabolite sets.

**Table 2:** Proportion of variance explained by genotype and metabolite components for five traits across different models (mean ± SD)

Trait	Genotypes only	Metabolites only			Genotype and Metabolites Combined		
	G	M1	M2	M1+M2	G+M1	G+M2	G+M1+M2
BF	0.36 ± 0.03	0.00 ± 0.00	0.11 ± 0.02	0.12 ± 0.02	0.36 ± 0.02	0.41 ± 0.03	0.38 ± 0.04
FCR	0.24 ± 0.03	0.02 ± 0.01	0.08 ± 0.01	0.08 ± 0.01	0.20 ± 0.03	0.24 ± 0.03	0.24 ± 0.02
DFI	0.27 ± 0.03	0.01 ± 0.01	0.11 ± 0.02	0.12 ± 0.02	0.26 ± 0.02	0.33 ± 0.02	0.30 ± 0.02
LD	0.41 ± 0.02	0.03 ± 0.01	0.03 ± 0.01	0.05 ± 0.01	0.40 ± 0.03	0.40 ± 0.03	0.41 ± 0.03
TDG	0.24 ± 0.03	0.01 ± 0.01	0.03 ± 0.01	0.03 ± 0.01	0.24 ± 0.02	0.25 ± 0.03	0.24 ± 0.03

For BF and DFI, the highest variance explained was achieved by the G+M2 model, indicating that M2 captures additional information beyond genotype. Similarly, TDG showed a slight improvement when metabolites were included, with G+M2 yielding the highest value. For LD and FCR, variance explained remained largely unchanged across G and G+M models.

### Prediction accuracy estimation

Table 3 presents a comparison of prediction accuracies for the five traits across multiple predictive models. Overall, the genotype-only model provided a strong baseline, whereas metabolite-only models generally showed lower prediction accuracy. Among metabolite-only models, M2 consistently outperformed M1 for BF, FCR, DFI, and TDG, while LD showed little difference between the two timepoints. Combining M1 and M2 improved prediction relative to M1 alone but provided little additional benefit over M2.

**Table 3:** Comparison of phenotype prediction accuracy for five traits across seven prediction models (Mean±sd)

Trait	Genotypes only	Metabolites only			Genotype and Metabolites Combined		
	G	M1	M2	M1+M2	G+M1	G+M2	G+M1+M2
BF	0.39 ± 0.03	0.06 ± 0.05	0.29 ± 0.06	0.29 ± 0.06	0.39 ± 0.03	0.46 ± 0.03	0.46 ± 0.04
FCR	0.27 ± 0.03	0.12 ± 0.05	0.25 ± 0.04	0.25 ± 0.03	0.27 ± 0.03	0.32 ± 0.03	0.32 ± 0.03
DFI	0.33 ± 0.04	0.08 ± 0.06	0.32 ± 0.05	0.32 ± 0.05	0.33 ± 0.03	0.42 ± 0.04	0.42 ± 0.04
LD	0.42 ± 0.03	0.16 ± 0.05	0.16 ± 0.05	0.19 ± 0.04	0.43 ± 0.03	0.43 ± 0.04	0.44 ± 0.04
TDG	0.30 ± 0.05	0.08 ± 0.03	0.13 ± 0.05	0.13 ± 0.04	0.30 ± 0.04	0.32 ± 0.04	0.32 ± 0.04

Integrating metabolite profiles with genotype data generally improved prediction accuracy over the genotype-only model, although the magnitude of improvement differed across traits. The greatest gains were observed when M2 was included with genotype data. For BF, FCR, DFI, and TDG, the G+M2 and G+M1+M2 models achieved the highest or near-highest accuracies and performed similarly. For LD, prediction accuracy was already high under the genotype-only model, and adding metabolite data led to a small improvement, with G+M1+M2 giving the highest value.

**Fig. 3:**
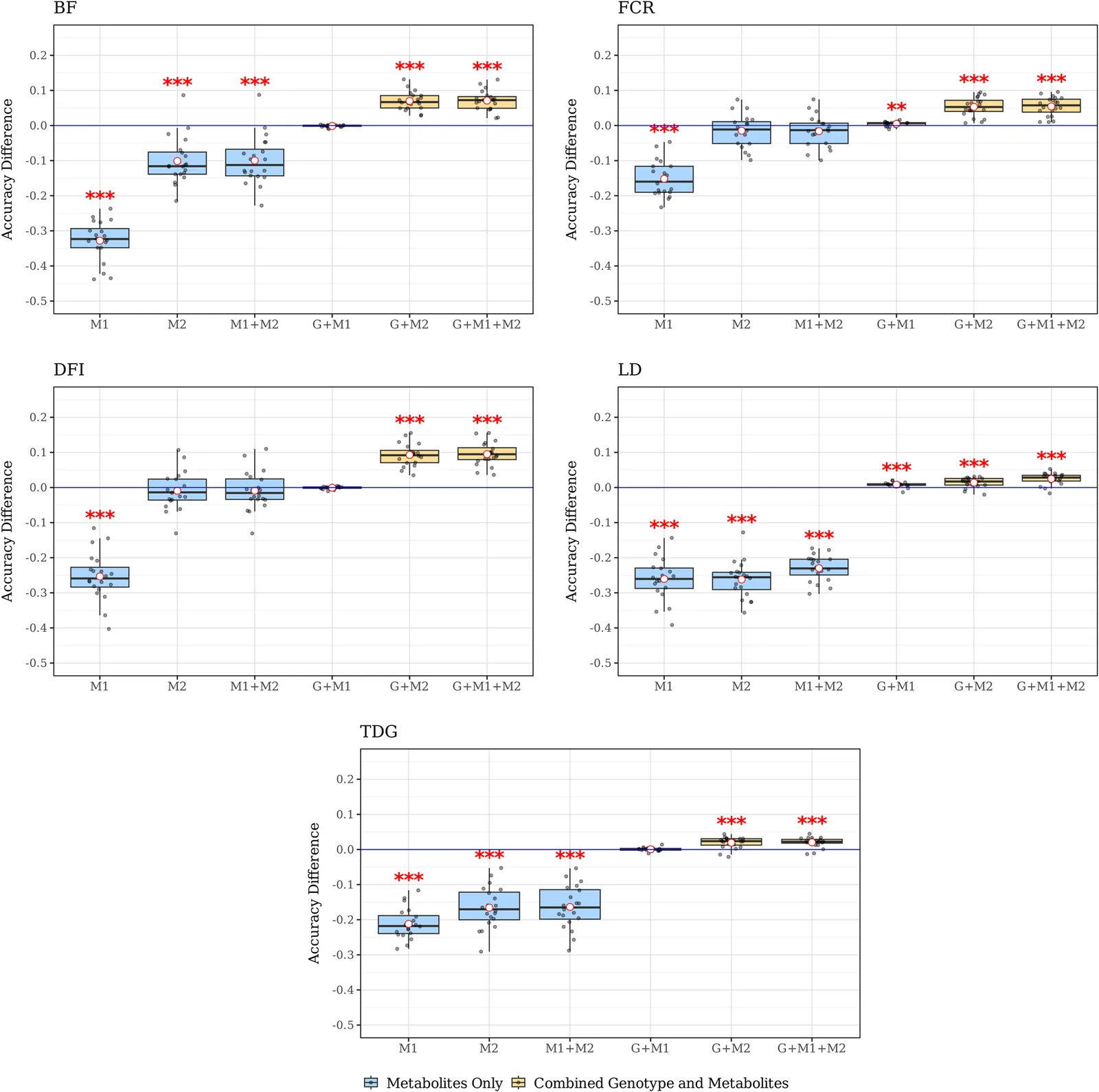
Box plots showing the differences in prediction accuracy across various models relative to the genotype-only baseline (Model G) for five traits. Each point represents, for one of 20 random train/test splits, the difference in prediction accuracy between the model indicated on the x-axis and the genotype-only (G) baseline. in these box plots, two distinct colors represent different model categories: blue indicates metabolites-only models (i.e., M1, M2, and M1+M2), and yellow indicates models combining genotypes and metabolites (i.e., G+M1, G+M2, G+M1+M2). Statistical significance is indicated by * (*p-value < 0.05, ** *p*-value < 0.01, *** *p*-value < 0.001)

Figure 3 shows the Monte Carlo cross-validation distribution of predictive accuracies for all models relative to G, used as the baseline, for all traits, in the form of box plots. These plots provide additional insight into how metabolite-profile data, either alone or in combination with genotype, impacts prediction accuracy relative to the genotype-only baseline.

All metabolite-only models generally yielded lower prediction accuracy than the genotype-only baseline for all traits. Significant reductions in accuracy (*p* < 0.001) were observed for BF, LD, and TDG in all metabolite-only models, and for FCR and DFI when only 10-week metabolite data (M1) were used. In contrast, for FCR and DFI, prediction accuracies from the 20-week metabolite model (M2) and the combined metabolite model (M1+M2) were not significantly different from the genotype-only model (*p* > 0.05). This suggests that late-stage metabolite-profile data alone may be sufficient to provide an alternative predictor for these two traits, with performance similar to using genomic data, when the latter data is not available. This finding highlights the potential utility of metabolite-profile data (especially late-stage) as indicators for specific economically important traits.

Furthermore, M2 showed a clear superiority over M1 as a single timepoint across traits, except for LD. Also, combining metabolite-profile data from both M1 and M2 yielded higher prediction accuracies than either timepoint alone, with some exceptions. This suggests that metabolite-profile data from different developmental stages capture complementary biological information, thus providing enhanced predictive ability.

Integrating metabolite-profile data with genotype information generally enhanced prediction accuracy, though the magnitude of the improvements varied depending on the trait and the age of metabolite sampling. Specifically, inclusion of 20-week metabolite-profile data G+M2 increased accuracy (*p* < 0.001) across all five traits, highlighting the importance of the late-stage metabolite-profile data. Incorporating only the 10-week metabolite-profile data (G+M1) did not improve prediction accuracy (*p* > 0.05) over the genotype-only model for DFI, BF, and TDG, whereas it provided noticeable gains for FCR (*p* < 0.01) and LD (*p* < 0.001). Combining metabolite-profile data from both timepoints (G+M1+M2) achieved a significant overall improvement in accuracy (*p* < 0.001) relative to the G model for all five traits. This indicates that including both early and late metabolite-profile data in the model allows it to explore complementary biological signals that are beneficial for the prediction of phenotypes.

The influence of each combination of type of information included in the model on the predictive performance may reflect how each trait has a distinct relationship dynamic with the blood metabolomic landscape. Notably, for DFI, M2 and M1+M2 performed similar to G, indicating that late-stage metabolite-profile data may serve as an alternative predictor. For FCR and LD, combining metabolite-profile data from either timepoint with genotype data improved prediction accuracy, with the highest gain achieved while using G+M2 and G+M1+M2. For LD, the greatest gain was observed when using both timepoints together with genotype data. This implies that these two traits might represent cumulative traits that benefit from metabolite information across multiple developmental stages. On the other hand, for BF and TDG, prediction accuracy differences between G and G+M1 were statistically non-significant (*p* > 0.05). However, for these two traits, both G+M2 and G+M1+M2 models provided the highest prediction accuracy, with a significance level of *p* < 0.001. All these patterns suggest that late-stage metabolite-profile data captured biologically more relevant information for predicting these five traits.

## Discussion

Our study evaluated the potential of integrating individual-level metabolite-profile data collected at multiple growth stages from blood serum into phenotype prediction models for five economically important traits in pigs: DFI, FCR, BF, TDG, and LD. Our findings confirm that many metabolites are moderately heritable, and combining them with genotype data led to consistent improvements in phenotype prediction accuracy across all traits. These findings align with previous studies, which reported moderate to high heritability for certain metabolites [18] and low to moderate heritability for several plasma metabolites in pigs [24]. In addition, our findings emphasize that the benefit of incorporating metabolite-profile data across different developmental stages into prediction models must be evaluated on a trait-by-trait basis. Importantly, integrating metabolite-profile data consistently complemented phenotypic predictions across all traits, consistent with previous findings that integrating metabolomic and other omics data with genomic information can improve phenotype prediction in pigs [25].

The biological relevance of incorporating metabolite-profile data in phenotypic prediction is trait dependent. For traits like DFI, the inclusion of late metabolite-profile data (20-week) provides further useful signals when combined with genotype data. Traits like LD, being influenced by both early and late biological stages, benefit from the integration of metabolite-profile data from multiple timepoints. These results are consistent with previous research showing that metabolite profiles are influenced by breed, nutritional environment, and physiological stage [26], which underscores the importance of aligning sampling strategy with traits. These findings support the targeted use of timepoint-specific metabolite-profile data for predicting the actual productive performance of individual pigs.

Heritability estimates for the metabolites ranged from low to moderate at both 10-week and 20-week timepoints. A similar range was reported for serum metabolites in dairy cattle, supporting the hypothesis that metabolite levels are genetically influenced [18]. Our analysis showed that heritability estimates varied across the 10-week and 20-week timepoints, while metabolite-profile data from 20 weeks were more useful for improving predictive performance when combined with genotypes for most traits. This aligns with previous findings that metabolite-profile data reflect distinct metabolic states and may be more informative for predictive performance at certain physiological stages [27]. In addition, genome-wide association studies and gene–metabolite network analyses have identified candidate biomarkers and biological pathways related to feed efficiency, further supporting the potential role of metabolite-profile data in improving trait prediction related to feed efficiency [28].

In the current analysis, we focused on phenotype prediction. Our results indicate that incorporating early-stage metabolite-profile data (10-week) may effectively capture biologically meaningful genetic variation that is useful in phenotypic prediction. Nonetheless, higher prediction accuracies were generally observed when incorporating late-stage (20-week) metabolite data. This pattern likely reflects the proximity of late-stage metabolite sampling to the actual phenotypic measurements, providing a more immediate snapshot of physiological status. In future studies, we plan to evaluate the utility of metabolite-profile data specifically for predicting breeding values, directly informing genetic improvement in livestock populations. Metabolites are genetically influenced intermediate phenotypes that can be linked to specific genomic loci through metabolite genome-wide association studies [28]. Identifying such loci would allow incorporation of metabolite-related biological information into genomic prediction models without requiring metabolite measurements for all selection candidates, thereby reducing costs and enabling early selection. In addition, multi-layer approaches such as neural network with mixed models (NNMM) [29] can explicitly model the genotype–metabolite–phenotype relationship and allow prediction for individuals with genotype data only by leveraging information from animals with metabolite records. Together, these strategies provide a framework for cost-effective omics-informed selection of complex traits.

## Conclusions

This study demonstrates that integrating individual-level metabolite-profile data with genotype data can improve the prediction accuracy for DFI, FCR, BF, TDG, and LD, which are key production traits in pigs. While metabolite-profile data alone yielded relatively low predictive power in most scenarios, likely due to the complex and temporally dynamic nature of the metabolome, their combination with genotype data consistently enhanced phenotype prediction performance across all traits. The moderate heritability observed for many metabolites further supports their potential as intermediate phenotypes that reflect both genetic and physiological processes. By integrating this omics data, prediction models gained access to non-redundant biological information not captured by genotypes alone. Future studies should focus on optimizing the timing and selection of metabolites, leveraging trait-specific biological knowledge, and integrating metabolomic information for the prediction of breeding values. Specifically, scenarios in which metabolite data are available only for a subset of animals should be explored using sophisticated modeling approaches, such as NNMM [29], to effectively incorporate partial metabolomic information.

## Electronic supplementary material

Below is the link to the electronic supplementary material.

Supplementary material 1

## Data Availability

The data used in this study are available from PIC upon reasonable request.

## References

[1] Forutan M, Lynn A, Aliloo H, Clark SA, McGilchrist P, Polkinghorne R, et al. Predicting phenotypes of beef eating quality traits. Front Genet. 2023;14:1089490. 10.3389/fgene.2023.1089490. PMC9936823 36816029

[2] Yin T, König S. Genomics for phenotype prediction and management purposes. Anim Front. 2016;6(1):65–72. 10.2527/af.2016-0010.

[3] Mrode R, Ojango JMK, Okeyo AM, Mwacharo JM. Genomic selection and use of molecular tools in breeding programs for indigenous and crossbred cattle in developing countries: current status and future prospects. Front Genet. 2019;9:694. 10.3389/fgene.2018.00694. PMC6334160 30687382

[4] Wiggans GR, VanRaden PM, Cooper TA. The genomic evaluation system in the United States: past, present, future. J Dairy Sci. 2011;94(6):3202–11. 10.3168/jds.2010-3866. 21605789

[5] Meuwissen THE, Hayes BJ, Goddard ME. Prediction of total genetic value using genome-wide dense marker maps. Genetics. 2001;157(4):1819–29. 10.1093/genetics/157.4.1819. PMC1461589 11290733

[6] Guan R, Li Y, Hu Z, Shi Y, Chen Z, Wu L, et al. Identifying slow-growing commercial pigs using growth performance and health indicators. Sci Rep. 2024;14(1):28222. 10.1038/s41598-024-78093-z. PMC11567956 39548132

[7] Delpuech E, Aliakbari A, Labrune Y, Fève K, Billon Y, Gilbert H, et al. Identification of genomic regions affecting production traits in pigs divergently selected for feed efficiency. Genet Sel Evol. 2021;53(1):49. 10.1186/s12711-021-00642-1. PMC8201702 34126920

[8] Legarra A, Christensen OF. Genomic evaluation methods to include intermediate correlated features such as high-throughput or omics phenotypes. JDS Commun. 2023;4(1):55–60. 10.3168/jdsc.2022-0276. PMC9873823 36713125

[9] Goldansaz SA, Guo AC, Sajed T, Steele MA, Plastow GS, Wishart DS. Livestock metabolomics and the livestock metabolome: a systematic review. PLoS One. 2017;12(5):e0177675. 10.1371/journal.pone.0177675. PMC5439675 28531195

[10] Fontanesi L. Metabolomics and livestock genomics: insights into a phenotyping frontier and its applications in animal breeding. Anim Front. 2016;6(1):73–79. 10.2527/af.2016-0011.

[11] Fiehn O. Metabolomics – the link between genotypes and phenotypes. Plant Mol Biol. 2002;48(1–2):155–71. 10.1023/A:1013713905833. 11860207

[12] Jang C, Hui S, Zeng X, Cowan AJ, Wang L, Chen L, et al. Metabolite exchange between mammalian organs quantified in pigs. Cell Metab. 2019;30(3):594–606.e3. 10.1016/j.cmet.2019.06.002. PMC6726553 31257152

[13] Banerjee P, Carmelo VAO, Kadarmideen HN. Integrative analysis of metabolomic and transcriptomic profiles uncovers biological pathways of feed efficiency in pigs. Metabolites. 2020;10(7):275. 10.3390/metabo10070275. PMC7408121 32640603

[14] Carmelo VAO, Banerjee P, da Silva Diniz WJ, Kadarmideen HN. Metabolomic networks and pathways associated with feed efficiency and related-traits in Duroc and landrace pigs. Sci Rep. 2020;10(1):255. 10.1038/s41598-019-57182-4. PMC6959238 31937890

[15] Guo X, Sarup P, Jahoor A, Jensen J, Christensen OF. Metabolomic-genomic prediction can improve prediction accuracy of breeding values for malting quality traits in barley. Genet Sel Evol. 2023;55(1):61. 10.1186/s12711-023-00835-w. PMC10478459 37670243

[16] Guo X, Sarup P, Bay Nord A, Henryon M, Ostersen T, Christensen OF. Metabolomic-genomic prediction realizes small increases in accuracy of estimated breeding values for daily gain in pigs. Genet Sel Evol. 2025;57(1):24. 10.1186/s12711-025-00972-4. PMC12096740 40399805

[17] Bromage TG, Idaghdour Y, Lacruz RS, Crenshaw TD, Ovsiy O, Rotter B, et al. The swine plasma metabolome chronicles “many days” biological timing and functions linked to growth. PLoS One. 2016;11(1):e0145919. 10.1371/journal.pone.0145919. PMC4703299 26735517

[18] Aliakbari A, Ehsani A, Vaez Torshizi R, Løvendahl P, Esfandyari H, Jensen J, et al. Genetic variance of metabolomic features and their relationship with body weight and body weight gain in Holstein cattle. J Anim Sci. 2019;97(9):3832–44. 10.1093/jas/skz228. PMC6735847 31278866

[19] Zhou X, Li C, He Z, Liu H, Wang M, He J. Metabolomics profiling of serum and urine from Chuanzang Black pigs with different residual feed intake. Anim (Basel). 2024;14(16):2323. 10.3390/ani14162323. PMC11350911 39199856

[20] Song B, Zheng C, Zheng J, Zhang S, Zhong Y, Guo Q, et al. Comparisons of carcass traits, meat quality, and serum metabolome between Shaziling and Yorkshire pigs. Anim nutr. 2022;8:125–34. 10.1016/j.aninu.2021.06.011. PMC8669263 34977382

[21] Hickey JM, Kinghorn BP, Tier B, van der Werf JHJ, Cleveland MA. A phasing and imputation method for pedigreed populations that results in a single-stage genomic evaluation. Genet Sel Evol. 2012;44(1):9. 10.1186/1297-9686-44-9. PMC3378429 22462519

[22] Cheng H, Fernando RL, Garrick DJ. JWAS: Julia implementation of whole-genome analysis software. Proceedings of the 11th World Congress on Genetics Applied to Livestock Production: 11–16 February 2018, Auckland. 2018.

[23] Wang Z, Cheng H. Single-trait and multiple-trait genomic prediction from multi-class Bayesian alphabet models using biological information. Front Genet. 2021;12:717457. 10.3389/fgene.2021.717457. PMC8542848 34707638

[24] Dervishi E, Yang T, Dyck MK, Harding JCS, Fortin F, Cheng J, et al. Heritability and genetic correlations of plasma metabolites of pigs with production, resilience and carcass traits under natural polymicrobial disease challenge. Sci Rep. 2021;11(1):20628. 10.1038/s41598-021-99778-9. PMC8526711 34667249

[25] Chen Y, Lonergan S, Lim KS, Cheng J, Dervishi E, Dyck MK, et al. Prediction of disease resilience of pigs using multi-omics data. J Anim Sci Biotechnol. 2026;17(1):107. 10.1186/s40104-026-01416-9. PMC13227672 42226240

[26] Liu Y, He Q, Azad MAK, Xiao Y, Yin Y, Kong X. Nuclear magnetic resonance-based metabolomic analysis reveals physiological stage, breed, and diet effects on the intramuscular metabolism of amino acids and related nutrients in pigs. Front Vet Sci. 2021;8:681192. 10.3389/fvets.2021.681192. PMC8382954 34447801

[27] Rohart F, Paris A, Laurent B, Canlet C, Molina J, Mercat MJ, et al. Phenotypic prediction based on metabolomic data for growing pigs from three main European breeds. J Anim Sci. 2012;90(13):4729–40. 10.2527/jas.2012-5338. 23100586

[28] Wang X, Kadarmideen HN. Metabolite genome-wide association study (mGWAS) and gene-metabolite interaction network analysis reveal potential biomarkers for feed efficiency in pigs. Metabolites. 2020;10(5):201. 10.3390/metabo10050201. PMC7281523 32429265

[29] Zhao T, Zeng J, Cheng H. Extend mixed models to multilayer neural networks for genomic prediction including intermediate omics data. Genetics. 2022;221(1):iyac034. 10.1093/genetics/iyac034. PMC9071534 35212766

