# Supplementary material for: Integrating longitudinal metabolite profiles improves phenotype prediction in pigs in a trait- and timepoint-dependent manner
Source: Genet Sel Evol. 2026 Oct 13;58:73. doi: 10.1186/s12711-026-01092-3 (PMC13660076; doi:10.1186/s12711-026-01092-3)
Supplement: Supplementary file 1 [file 12711_2026_1092_MOESM1_ESM.pdf]

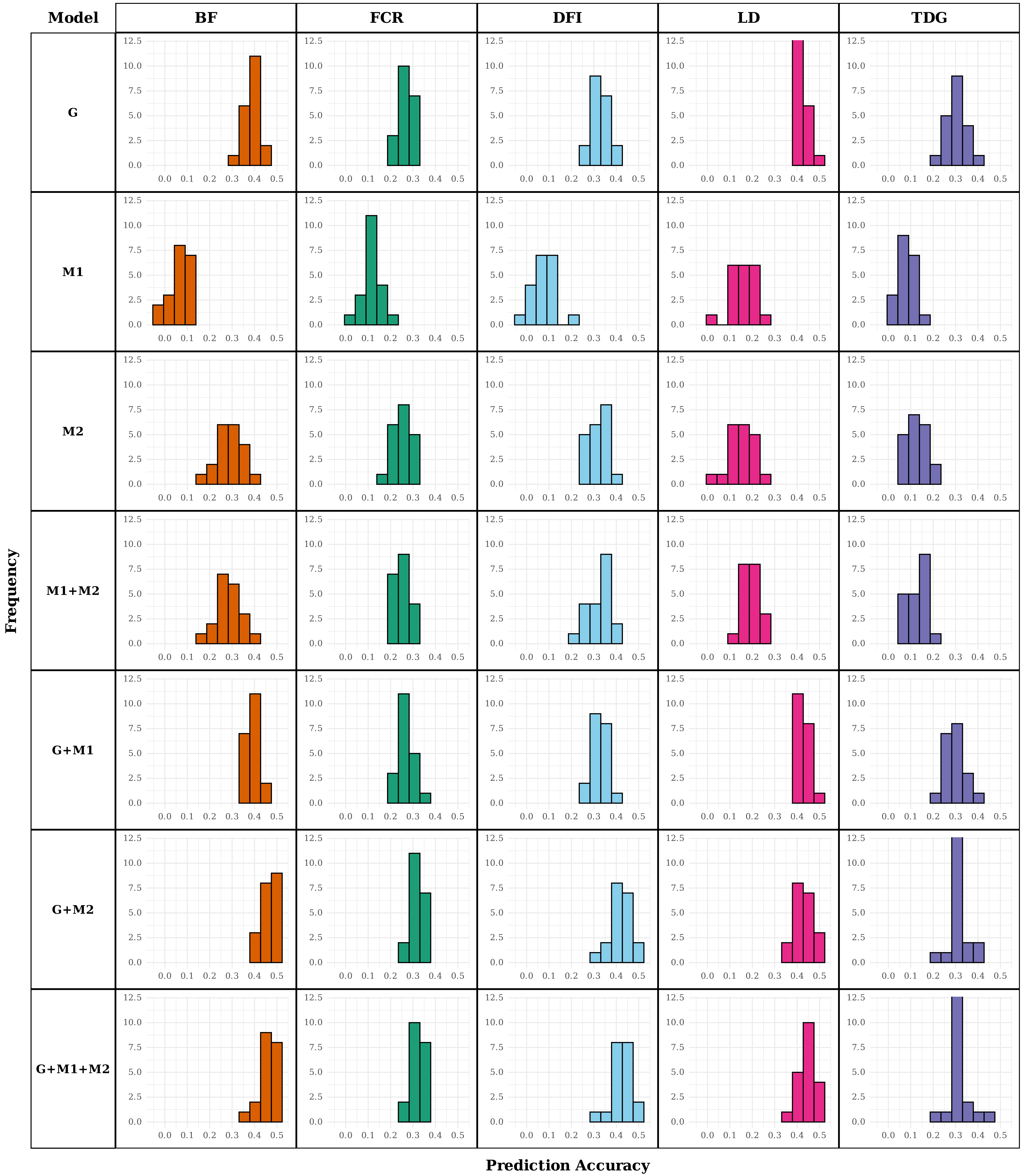
Model
BF
FCR
DFI
LD
TDG
G
M1
M2
M1+M2
G+M1
G+M2
G+M1+M2
Frequency
Prediction Accuracy
